# Relationship Between Psychiatric Drug Use and Cognitive Function in Women During the Menopausal Transition: An Analysis Based on the NHANES Database

**DOI:** 10.62641/aep.2352

**Published:** 2026-10-15

**Authors:** Yan Jiang, Hui Sun, Hui Ma, Chenyang Zhao

**Affiliations:** ^1^Department of Pharmacy, The Second Hospital of Dalian Medical University, 116023 Dalian, Liaoning, China

**Keywords:** psychotropic drugs, polypharmacy, cognitive risk, menopausal transition

## Abstract

**Background:**

Women during the menopausal transition often experience mood disorders, sleep disturbances, and cognitive decline, making the use of psychotropic drugs relatively common. However, the relationship between psychiatric drug use and cognitive function in menopausal women remains unclear.

**Methods:**

This was a cross-sectional study that included women aged 45–65 years from the National Health and Nutrition Examination Survey (NHANES) 2021–2023 cycle. Psychiatric drug use was defined based on prescription drug data, including antidepressants, benzodiazepines, and sedative-hypnotics. Cognitive function was assessed using the Digit Symbol Substitution Test (DSST). The Bayesian Kernel Machine Regression model was used to analyse the relationship between psychiatric drug use and cognitive function, with adjustment for potential confounders, including routine demographic characteristics.

**Results:**

A total of 956 women during the menopausal transition were included in the analysis. As the number of psychotropic drugs used increased, cognitive function decline became more pronounced. Antidepressants and benzodiazepines played dominant roles in this association (posterior inclusion probability >0.9). The concurrent use of two or more psychotropic drugs was associated with an 11%–24% reduction in DSST cognitive scores. In the context of three-drug combinations, the cognitive impairment associated with antidepressants and benzodiazepines were amplified, resulting in an additional 5.1%–6.3% decrease in cognitive scores.

**Conclusions:**

Psychiatric medication use showed a significant cross-sectional association with poorer cognitive performance among women undergoing menopausal transition, and polypharmacy regimens displayed stronger associations than single-drug regimens. Clinically, greater attention should be paid to the potential cognitive impact of psychiatric drug use in menopausal women, and polypharmacy regimens should be optimised.

## Introduction

The menopausal transition is a critical physiological period during which women shift from the reproductive stage to the postmenopausal phase, primarily characterised by ovarian function decline and fluctuations in oestrogen levels [1]. Some studies have shown that women undergoing the menopausal transition are often accompanied by symptoms such as hot flashes, sleep disturbances, mood swings, depression, and anxiety, and may also experience cognitive changes, such as decreased attention and impaired memory [2, 3, 4]. In the process of global population aging, cognitive health issues in women undergoing the menopausal transition are gradually receiving widespread attention. Existing epidemiological studies have shown that the prevalence of clinically significant depressive symptoms in women undergoing the menopausal transition is several times higher than that in premenopausal women [5, 6].

Because women undergoing the menopausal transition often experience mental and psychological symptoms such as insomnia, anxiety, and depression, psychiatric drug use is relatively common in this population. Antidepressants, benzodiazepines, and sedative-hypnotic drugs have become common treatment drugs for improving menopausal-related symptoms [7, 8]. Since the menopausal transition usually lasts for several years, women are very likely to have long-term use of psychotropic drugs, which may potentially affect their neurocognitive function. Previous studies have shown that some psychotropic drugs may have adverse effects on cognitive function through mechanisms such as affecting central neurotransmission, increasing anticholinergic load, and inhibiting neural activity [9, 10]. Among them, tricyclic antidepressants with anticholinergic effects may cause memory loss and cognitive decline by interfering with cholinergic neurotransmission; some antidepressants may also cause drowsiness and psychomotor retardation [11, 12, 13]. However, current research on the relationship between long-term medication use and cognitive function are still inconsistent.

A study suggests that antidepressant treatment may have certain beneficial effects on cognitive function after alleviating depressive symptoms [14]. Overall, with increasing psychosocial stress and improved access to mental health services, psychotropic drug use among middle-aged and older women shows a gradually rising trend [15]. Additionally, women undergoing the menopausal transition are more likely to use multiple psychotropic drugs concurrently rather than just a single drug, which may indicate potential synergistic or additive effects between different drugs [16]. Traditional statistical methods usually evaluate only the linear relationship between a single drug and an outcome, making it difficult to fully reflect the complex effects of multiple psychotropic drug exposures on cognitive function. Therefore, statistical methods that can address nonlinear relationships and interactions are needed to further explore the relationship between multiple psychotropic drug exposure and cognitive function.

Based on the National Health and Nutrition Examination Survey (NHANES) database, this study investigates the relationship between psychotropic drug use and cognitive function in women during the menopausal transition. The Bayesian Kernel Machine Regression (BKMR) model was applied to assess the joint associations, nonlinear relationships, and potential interactions of multiple psychotropic drug exposures in relation to cognitive function, aiming to provide epidemiological evidence for the rational use of psychotropic drugs and the protection of cognitive function in women undergoing the menopausal transition.

## Materials and Methods

### Study Population

This study used data from the NHANES 2021–2023 cycle. Since this study only analysed de-identified publicly available data, additional ethical approval was not required. A total of 4879 women aged 45–65 years were initially identified. Among them, 1293 had completed the Digit Symbol Substitution Test (DSST). After excluding 105 individuals with a history of neurological or psychiatric disorders, 63 with missing covariate data, and 169 with incomplete prescription drug records, 956 eligible women were finally included in the final analysis. Baseline characteristics were compared between the included and excluded participants. No significant differences were observed in demographic, except for smoking status (**Supplementary Table 1**), suggesting that excluding these participants would not introduce substantial selection bias.

### Included Criteria

(1) female; (2) aged 45–65, corresponding to the menopause-related age range [17]; (3) completed cognitive function tests; (4) drug exposure status was determined using the NHANES prescription medication questionnaire, which records prescription medications used during the 30 days preceding the interview. Participants reporting at least one psychotropic medication during this period were classified as psychotropic medication users, whereas those without any reported psychotropic medication use during the same period served as the reference group; and (5) a record of prescription drug use must have at least one registration of psychotropic drug exposure information.

This study focuses on three types of psychotropic drugs. The drug variables were screened and matched based on NHANES official drug codes and generic names (Table 1): (1) antidepressants, including selective serotonin reuptake inhibitors (e.g., fluoxetine, paroxetine, and sertraline), serotonin–noradrenaline reuptake inhibitors (e.g., venlafaxine and duloxetine), and tricyclic antidepressants (e.g., amitriptyline and doxepin); (2) benzodiazepines, including alprazolam, lorazepam, diazepam, and clonazepam; and (3) sedative-hypnotics, including Z-drugs (zolpidem, eszopiclone, zaleplon, and zopiclone).

**Table 1.** **Official RxNorm codes for the 15 psychotropic drugs based on NHANES**.

Drug	RxNorm
Fluoxetine	3127
Paroxetine	3129
Sertraline	3130
Venlafaxine	3132
Duloxetine	28444
Amitriptyline	2140
Doxepin	2142
Alprazolam	2035
Lorazepam	2041
Diazepam	2037
Clonazepam	2036
Zolpidem	3440
Eszopiclone	68727
Zaleplon	3439
Zopiclone	10491

NHANES, National Health and Nutrition Examination Survey.

### Exclusion Criteria

(1) Previously self-reported stroke, Parkinson’s disease, dementia, or other serious neurological diseases; (2) Previously diagnosed psychiatric disorders, including schizophrenia, bipolar disorder, and other serious mental disorders; (3) Missing key covariates, including body mass index (BMI), education level, race, and smoking (Definition: at least 100 cigarettes during their lifetime), and alcohol consumption (Definition: ≥12 drinks/year); and (4) Taking drugs (excluding the study target drugs) that may affect mood or mental state within the past 30 days.

### Cognitive Function Assessment

Cognitive function was assessed using the DSST from the NHANES. This test is a sensitive measure of executive function and information processing speed, and has been widely used for the quantitative assessment of cognitive function in epidemiological studies. Higher scores indicate better cognitive performance [18]. The DSST was used both as a continuous variable and as a four-level categorical variable based on quartiles (≤29; 30–39; 40–48; 49+), the specific scoring criteria were applied as previously described [19].

### Statistical Analysis

All statistical analyses were performed using R software (version 4.2.1, R Foundation for Statistical Computing, Vienna, Austria). Continuous variables were presented as mean ± standard deviation, and categorical variables were presented as frequency (percentage) [n (%)]. Normality of continuous variables was tested using the Shapiro–Wilk test, and homogeneity of variances was tested using Levene’s test. For continuous variables that followed a normal distribution and had homogeneous variances, comparisons among multiple groups were performed using ANOVA. Comparisons of categorical variables between groups were performed using the chi-square test or Fisher’s exact test, as appropriate.

The BKMR model implemented in the R package was used, with different types of psychotropic drugs treated as mixture exposure variables. In this study, psychiatric drug use was modelled as a four-level categorical variable (no drug use; use of one type; use of two types; use of three types), cognitive test score was used as the outcome variable, and adjustments were made for covariates including age, race, education level, BMI, smoking, and alcohol use. The BKMR analysis primarily assessed: (1) the overall effect of the psychiatric drug mixture; (2) the independent association of each drug with cognitive function; (3) potential interactions between different drug types; and (4) nonlinear exposure–response relationships. The model was fitted using 50,000 iterations, with the first 10,000 iterations discarded as burn-in, and the remaining 40,000 iterations used for posterior distribution estimation. A posterior inclusion probability (PIP) closer to 1 indicates stronger evidence that the corresponding drug type contributes to the mixture effect.

## Results

### Baseline Characteristics of Participants

The baseline characteristics of the 956 women included in the study are presented in Table 2. The mean age of participants was 53.4 ± 7.7 years. The mean BMI was 29.1 ± 6.7 kg/m^2^. Most participants had at least a high school education, and Non-Hispanic White individuals constituted the largest racial/ethnic group. Approximately two-thirds of participants reported alcohol consumption, whereas 30.0% reported smoking. Our analysis showed that the mean DSST score was 39.6 ± 13.7, and DSST scores tended to decrease with increasing age (*p* < 0.001). Regarding psychiatric drug use, 86.9% of participants reported the use of at least one type of psychotropic drugs, and 49.0% reported polypharmacy (concurrent use of multiple drugs).

**Table 2.** **Baseline characteristics of women from the NHANES database (n = 956)**.

Variable		Overall	45–50 years old	51–60 years old	≥61 years old	F/χ^2^	*p*
		(n = 956)	(n = 322)	(n = 582)	(n = 52)		
Age (years)		53.4 ± 7.7	47 ± 2.3	56.3 ± 2.9	62.3 ± 1.6	726.8	<0.001
DSST score		39.6 ± 13.7	44.5 ± 13.8	37.2 ± 11.9	30.9 ± 11.8	86.3	<0.001
DSST group						61.52	<0.001
	≤29	218 (22.8%)	52 (16.1%)	148 (25.4%)	18 (34.6%)		
	30–39	275 (28.8%)	89 (27.6%)	166 (28.5%)	20 (38.5%)		
	40–48	302 (31.6%)	110 (34.2%)	179 (30.8%)	13 (25.0%)		
	49+	161 (16.8%)	71 (22.0%)	89 (15.3%)	1 (1.9%)		
BMI (kg/m^2^)		29.1 ± 6.7	28.4 ± 6.3	29.7 ± 6.8	28.6 ± 5.9	2.1	0.08
Smoking status (yes)		287 (30.0%)	183 (56.8%)	84 (14.4%)	20 (38.5%)	136.5	<0.001
Alcohol consumption (yes)		650 (68.0%)	222 (68.9%)	392 (67.4%)	36 (69.2%)	0.3	0.03
Education level						42.7	<0.001
	Below high school	63 (6.6%)	25 (7.8%)	20 (3.4%)	18 (34.6%)		
	High school/GED	681 (71.2%)	199 (61.8%)	459 (78.9%)	23 (44.2%)		
	College or above	212 (22.2%)	98 (30.4%)	103 (17.7%)	11 (21.2%)		
Race						10.31	0.58
	Non-Hispanic White	571 (59.7%)	182 (56.5%)	356 (61.2%)	33 (63.5%)		
	Non-Hispanic Black	115 (12.0%)	42 (13.0%)	66 (11.3%)	7 (13.5%)		
	Hispanic	106 (11.1%)	48 (14.9%)	52 (8.9%)	6 (11.5%)		
	Other	164 (17.2%)	50 (15.6%)	108 (18.6%)	6 (11.5%)		
Number of psychotropic drugs						28.92	<0.001
	0	125 (13.1%)	46 (14.3%)	72 (12.4%)	7 (13.5%)		
	1	362 (37.9%)	128 (39.8%)	219 (37.6%)	15 (28.8%)		
	2	328 (34.3%)	101 (31.4%)	204 (35.1%)	23 (44.2%)		
	3	141 (14.7%)	47 (14.6%)	87 (14.9%)	7 (13.5%)		

Note: DSST, Digit Symbol Substitution Test; BMI, body mass index; GED, General Educational Development; NHANES, National Health and Nutrition Examination Survey.

### Overall Association Between Combined Use of Three Types of Psychotropic Drugs and DSST Scores

BKMR analysis of polypharmacy (Fig. 1) revealed a nonlinear negative association between combined use of psychotropic drugs and DSST scores in women. At low exposure levels (use of 0–1 type drug), no statistically significant association with cognitive function was observed. However, when the exposure level exceeded the 0.5 quantile (i.e., concurrent use of two or more type of psychotropic drugs), DSST scores decreased significantly, suggesting a potential synergistic cognitive impairment effect of polypharmacy. Specifically, at low exposure levels, the estimated association with DSST scores was close to zero. Compared with non-users, women using a single type of psychiatric drug showed a mean decrease in DSST score of less than 3%, which was not statistically significant. When exposure exceeded the 0.5 quantile, DSST scores declined by approximately 11% compared with those using two type drugs, with the 95% confidence interval lying entirely below zero, indicating statistical significance. As the overall exposure level further increased to the 0.7 quantile, the reduction in DSST scores reached 24%, demonstrating a markedly amplified synergistic impairment effect of polypharmacy.

**Fig. 1.**
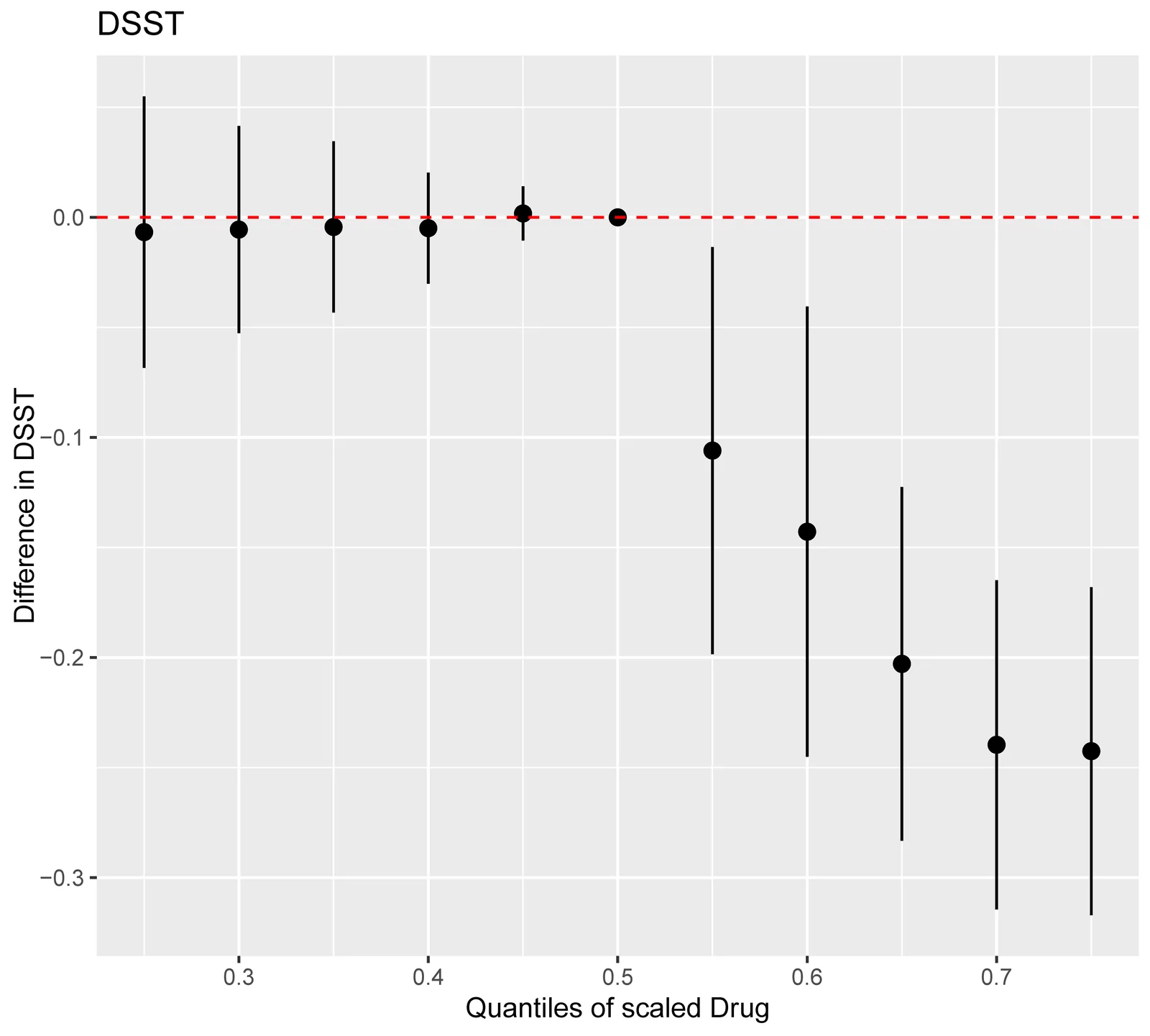
**Total effects of three types of psychotropic drugs on cognitive function (DSST) in women**. Note: Adjusted for age, BMI, education level, race, smoking, and drinking. DSST, Digit Symbol Substitution Test.

The PIP results from the BKMR model (Table 3) showed that benzodiazepines (PIP = 0.980) and antidepressants (PIP = 1.000) were the primary exposure factors influencing DSST-measured cognitive function in women, indicating potential independent associations. In contrast, the PIP for sedative-hypnotics was only 0.092, suggesting no significant independent association with cognitive function.

**Table 3.** **Posterior inclusion probabilities (PIPs) of three types of psychotropic drugs for DSST cognitive function in women**.

Variable	Group	PIP
Antidepressants	1	1
BZDs	2	0.98
Sedative‑hypnotics	3	0.092

Note: Adjusted for age, BMI, education level, race, smoking, and drinking. DSST, Digit Symbol Substitution Test; BZDs, benzodiazepines.

### Independent Associations Between Individual Psychotropic Drug Types and Cognitive Function

The BKMR univariate exposure–response analysis (Fig. 2) revealed that benzodiazepines showed the strongest association with DSST-measured cognitive function in women, significantly reducing cognitive processing speed even at low-to-moderate exposure levels. In contrast, antidepressants and sedative-hypnotics, when used alone, showed no significant independent association with cognitive function in this study. The positive fluctuation observed for benzodiazepines at high exposure levels should be interpreted with caution due to the limited sample size at extreme exposure levels, and may not have practical biological significance.

**Fig. 2.**
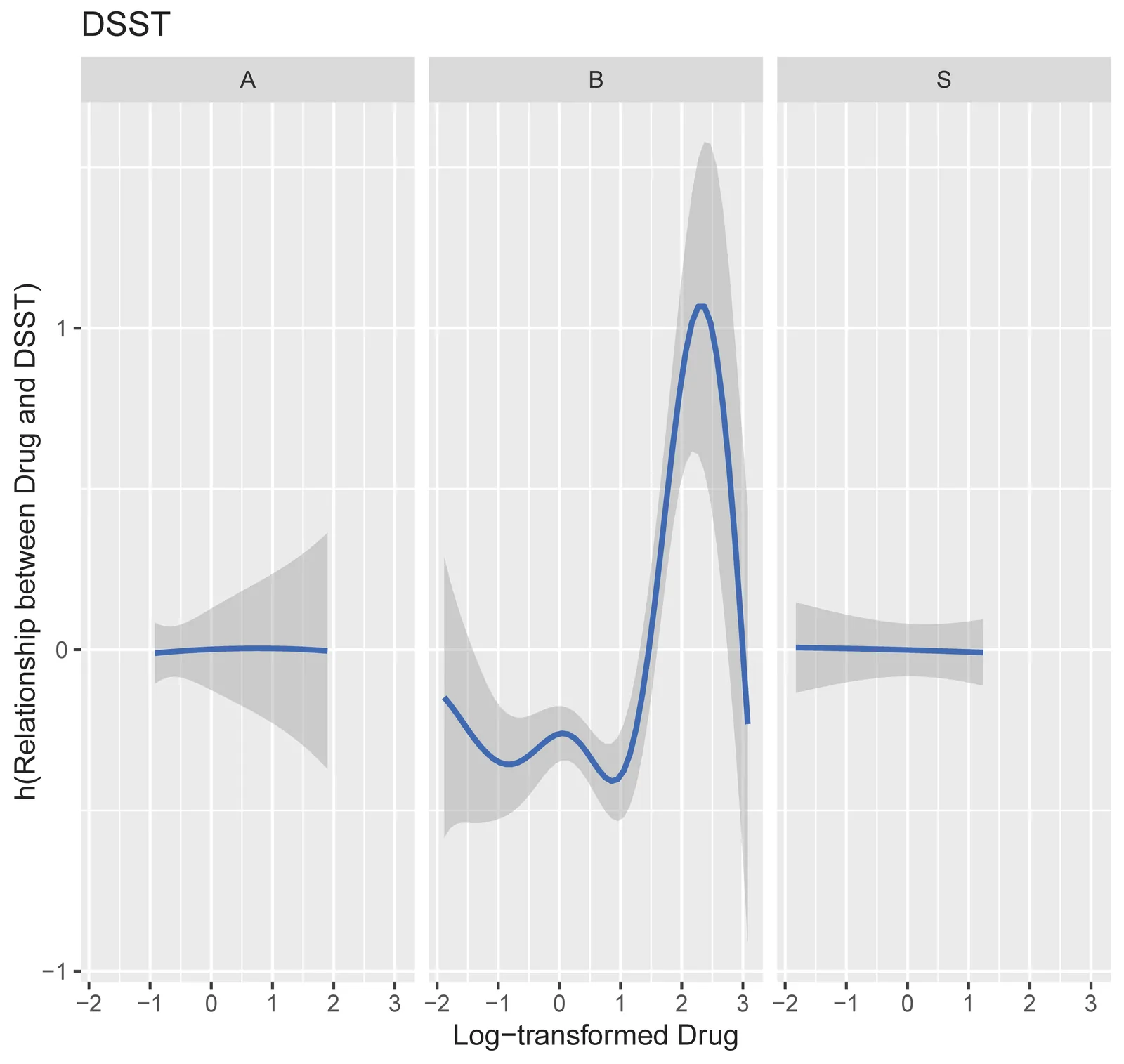
**Single-drug exposure–response relationships between three types of psychotropic drugs and DSST cognitive function in women**. Note: A, antidepressants; B, benzodiazepines; S, sedative-hypnotics. The 0.25, 0.5, and 0.75 quantiles do not represent dose quantiles of individual drugs. Instead, they represent different levels of overall psychotropic polypharmacy burden, defined by the number of concurrently used psychotropic medication classes (0, 1, 2, or 3 types), reflecting different backgrounds of combined medication use. Adjusted for age, BMI, education level, race, smoking, and drinking. DSST, Digit Symbol Substitution Test.

### Interactions Among Three Types of Psychotropic Drugs in Relation to Cognitive Function in Women

In Fig. 3, antidepressants showed associations with lower DSST scores across different polypharmacy levels. Among participants using one type (low-exposure), antidepressant use was associated with 1.2% score reduction, with confidence intervals crossing zero. Similarly, among those using two types of psychotropic drugs (moderate exposure), antidepressant use was associated with 2.5% score reduction, with confidence intervals crossing zero. The most severe cognitive decline (6.3% reduction, confidence intervals not crossing zero) was detected in the three-drug combination group (high exposure).

**Fig. 3.**
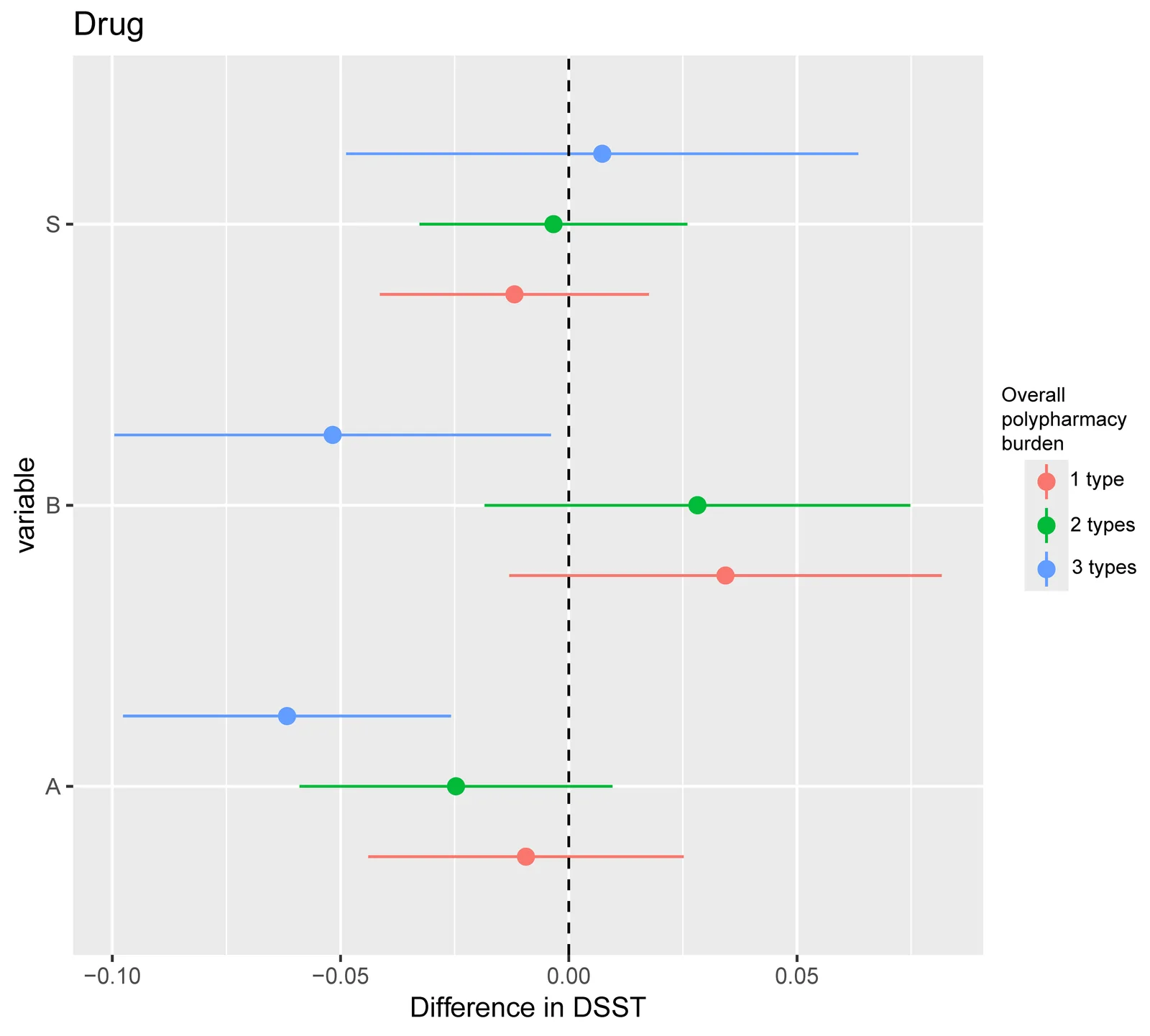
**Interactions among three types of psychotropic drugs in relation to DSST cognitive function in women**. Note: A, antidepressants; B, benzodiazepines; S, sedative-hypnotics. Adjusted for age, BMI, education level, race, smoking, and drinking. DSST, Digit Symbol Substitution Test.

For benzodiazepines, monotherapy and dual-drug co-use significantly improved DSST scores by 2.6% and 3.0%, respectively, although the confidence intervals crossed zero. These findings indicated no independent cognitive impairment at low or moderate polypharmacy burden. This cognitive benefit was not observed in triple-drug regimens, in which cognitive performance dropped by 5.1% and the confidence intervals did not cross zero. Sedative-hypnotics triggered a 1.3% decline in DSST scores when used alone, although the confidence interval crossed zero; no statistically significant associations were observed for co-use of two or three types of psychotropic drugs (0% score change).

Across all polypharmacy strata, only antidepressants induced progressively worse cognitive outcomes as the number of concurrent psychiatric drug use increase, with their harmful cognitive effects amplified under three-drug combination. Benzodiazepines only impaired cognition when paired with two additional psychiatric medications.

## Discussion

This study systematically analysed the effects of individual and combined exposure to three types of psychotropic drugs—antidepressants, benzodiazepines, and sedative-hypnotics—on DSST scores in women using the BKMR model. This study provides evidence that polypharmacy of psychotropic drugs, rather than individual medication use alone, may represent an important factor associated with cognitive performance among middle-aged and older women. By evaluating the combined burden of multiple psychotropic medication classes, our findings extend previous research that has primarily focused on single-drug exposure.

The observed association between concurrent benzodiazepine and antidepressant use and poorer cognitive performance highlights the potential importance of considering medication combinations in clinical cognitive risk assessment. Although the underlying mechanisms remain to be elucidated, the findings may be biologically plausible given the distinct effects of these medications on central nervous system pathways involved in attention, information processing, and executive function. The results revealed that benzodiazepine use was associated with poorer cognitive performance in women. Moreover, we identified strong associations between concurrent use of these medications and poorer cognitive performance, forming a notable association pattern observed in women with lower DSST scores. From a mechanistic perspective, benzodiazepines act on the central nervous system and may affect executive functions such as attention and information processing speed, which is consistent with the finding that their use alone has been associated with cognitive impairment [20, 21]. In contrast, antidepressants used alone may exert a neutral or even mildly protective effect by improving mood and alleviating depression-related cognitive impairment [22, 23]. However, cognitive impairment is induced only when antidepressants are used in combination with sedative agents such as benzodiazepines, because of potential additive effects on central nervous system depression. In this study, sedative-hypnotics did not exhibit independent cognitive impairment effects, which may be attributed to the fact that commonly used non-benzodiazepine hypnotics in clinical practice have short half-lives and minimal daytime sedative side effects [24]. However, these drugs may amplify the cognitive impairing effects of other drugs in the context of polypharmacy, demonstrating a potential interaction.

The findings of this study further suggest that women face a risk of cognitive impairment associated with polypharmacy in the context of psychiatric drug use. Compared with monotherapy, the combined use of benzodiazepines and antidepressants poses a more pronounced hazard to cognitive function, potentially producing additive effects at the level of the central nervous system through distinct mechanisms of action, thereby exerting more substantial adverse effects on cognitive functions such as attention, information processing speed, and executive function. Previous studies have demonstrated that benzodiazepines, by enhancing γ-aminobutyric acid-mediated central inhibition, reduce neural activity in the prefrontal cortex and hippocampus, leading to psychomotor slowing and impaired memory processing. Meanwhile, some antidepressants, particularly those with anticholinergic properties, may also affect cholinergic neurotransmission, thereby interfering with attention and learning and memory processes [25, 26]. Considering the high prevalence of psychiatric symptoms in women during the perimenopausal and postmenopausal stages [27], psychiatric drug use and polypharmacy are relatively common in clinical practice, which may further amplify the risk of cognitive impairment in this population. In clinical practice, the use of benzodiazepines alone should be carefully managed, and the necessity of polypharmacy among women should be rigorously evaluated. Long-term concurrent use of antidepressants and benzodiazepines should be avoided whenever possible to reduce the potential risk of cognitive impairment. Furthermore, although sedative-hypnotics showed no association with poorer cognitive performance in this study, they should still be used with caution in polypharmacy regimens to avoid additive adverse effects of combined medications.

Several limitations of this study should be acknowledged. Because participants with missing cognitive assessment or prescription medication data were excluded, selection bias cannot be completely excluded. After stratifying by age, the sample distribution was uneven, as follow-up and inclusion of older individuals are more challenging—a common issue in the NHANES database as well. Some categorical variables had very small frequencies; although appropriate statistical methods were applied, residual bias may still exist. Second, this was a cross-sectional observational study, which precludes establishing causal relationships. Additionally, detailed information regarding specific drug types, dosages, or durations of use was unavailable. Future longitudinal cohort studies are warranted to further validate the long-term effects of psychiatric drug polypharmacy on cognitive function in women, particularly in older age groups.

## Conclusions

This study found that benzodiazepine use alone was associated with poorer cognitive impairment in women, whereas antidepressants showed stronger associations with cognitive performance when used in combination with other drugs. The polypharmacy pattern combining benzodiazepines and antidepressants was positively associated with poorer cognitive performance among women. Sedative-hypnotics showed no significant independent association with cognitive function in this study, although they may synergistically increase the risk of cognitive impairment in the context of polypharmacy. Clinically, attention should be given to managing the use of benzodiazepines and polypharmacy involving psychotropic drugs.

## Availability of Data and Materials

The datasets used during the current study were available from the corresponding authors on reasonable request.
