# Supplementary material for: Relationship Between Psychiatric Drug Use and Cognitive Function in Women During the Menopausal Transition: An Analysis Based on the NHANES Database
Source: Actas Esp Psiquiatr. 2026 Oct 15;54(5):1657–65. doi: 10.62641/aep.2352 (PMC13714489; doi:10.62641/aep.2352)
Supplement: Supplementary file 1 [file ActEsp-54-5-1657-1665-s1.zip › AEP-2352-Supplementary materials.docx]

Table S1 Baseline characteristics of included and excluded participants.

| **Variable** | Included (n=956) | Excluded (n=3923) | *P* |
| --- | --- | --- | --- |
| **Age (years)** | 53.44 ± 7.70 | 52.83 ± 8.11 | 0.072 |
| **BMI (kg/m^2^)** | 29.13 ± 6.71 | 29.69 ± 7.02 | 0.063 |
| **Smoking（yes）** | 287(30.0%) | 1805 (46.0%) | ＜ 0.001 |
| **Drink（yes）** | 650(68.0%) | 2601 (66.3%) | 0.326 |
| **Education level** |  |  | 0.214 |
| < high school | 63(6.6%) | 294 (7.5%) |  |
| high school / GED | 681(71.2%) | 2758 (70.3%) |  |
| college or above | 212(22.2%) | 871 (22.2%) |  |
| **Race** |  |  | 0.387 |
| Non-Hispanic White | 571(59.7%) | 2295 (58.5%) |  |
| Non-Hispanic Black | 115(12.0%) | 502 (12.8%) |  |
| Hispanic | 106(11.1%) | 451 (11.5%) |  |
| other | 164(17.2%) | 675 (17.2%) |  |

BMI, body mass index; GED, General Educational Development (a high school equivalency credential).
